# Lactic acid bacteria: A sustainable solution against phytopathogenic agents

**DOI:** 10.1111/1758-2229.70021

**Published:** 2024-12-02

**Authors:** Andreia Saragoça, Henrique Canha, Carla M. R. Varanda, Patrick Materatski, Ana Isabel Cordeiro, José Gama

**Affiliations:** ^1^ Biosciences School of Elvas Polytechnic Institute of Portalegre Elvas Portugal; ^2^ MED—Mediterranean Institute for Agriculture, Environment and Development, & CHANGE—Global Change and Sustainability Institute, Institute for Advanced Studies and Research Pólo da Mitra, Ap. 94 Évora 7006‐554 Portugal; ^3^ Research Centre for Natural Resources, Environment and Society (CERNAS), Santarém Polytechnic University, School of Agriculture Quinta do Galinheiro ‐ S. Pedro Santarém 2001‐904 Portugal; ^4^ VALORIZA—Centro de Investigação para a Valorização de Recursos Endógenos Portalegre Portugal

## Abstract

Biological control agents (BCAs) are beneficial living organisms used in plant protection to control pathogens sustainably. Lactic acid bacteria (LAB) have gained attention in biopesticides due to their safety as recognized by the Food and Drug Administration. These bacteria possess antifungal properties, demonstrating inhibitory effects through nutrient competition or the production of antimicrobial metabolites. Numerous *Lactobacillus* species have shown the ability to inhibit pathogenic microorganisms, primarily through acid production. The organic acids secreted by LAB reduce the pH of the medium, creating a hostile environment for microorganisms. These organic acids are a primary inhibition mechanism of LAB. This article reviews several studies on LAB as BCAs, focusing on their inhibition modes. Additionally, it discusses the limitations and future challenges of using LAB to control phytopathogens for sustainable agriculture.

## Graphical abstract

This review highlights the role of lactic acid bacteria as biological control agents and discusses their potential as alternative strategies to control phytopathogens towards a sustainable agriculture. LAB have a long history in food science and present the GRAS status, making them good candidates for applications in plant protection. LAB limitations and challenges that have prevented their application in agriculture are also discussed.

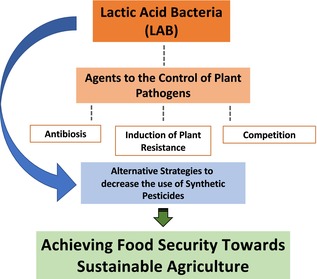

## INTRODUCTION

Currently, the agricultural sector faces numerous challenges, due to the growing population and the need to feed more than 8 billion people becoming a demanding global concern. In addition to this, the field is facing issues relating to climate change, such as floods and droughts, and emerging phytopathogenic agents, which cause significant losses in crops (Omran & Baek, 2022). Over the past 300 years, agricultural systems have expanded and now account for approximately 40% of the Earth's land surface (Foley et al., 2005). However, it is estimated that between 10% and 30% of food production is lost worldwide, due to phytopathogens, jeopardizing food security (Agrios, 2005). These pathogens also endanger food safety: directly, through the toxins they produce and that are harmful to humans and animals through the ingestion of contaminated plants (Patel et al., 2022), and indirectly through the chemical products used for their control. Fungi of the genera *Aspergillus*, *Fusarium*, *Penicillium* and *Alternaria* are among the fungi that produce toxins and present a risk of food contamination (Wagacha & Muthomi, 2008).

Among important phytopathogens, *Phytophthora infestans* left its mark in history after, in 1840, having caused significant cultural and economic consequences, including starvation which resulted in the death of approximately 1 million people (Omran & Baek, 2022). *Fusarium* spp. is also among the most economically important pathogens, causing wilts and rots on a wide range of crops and producing mycotoxins harmful to humans and animals (Johns et al., 2022). *Alternaria* spp. are among the most ubiquitous fungi, these fungi cause leaf spot diseases in many crops (Hou et al., 2016), with *A. alternata* being one of the most common species (Nowicki et al., 2022). Fungal plant pathogens from *Colletotrichum* genera are also responsible for serious losses in several crops such as olive (Materatski et al., 2019). Other phytopathogens, such as *Claviceps* sp., are responsible for great losses in various cereal crops and can cause ergotism in humans, leading to peripheral sensation loss, hallucinations or even death (Omran & Baek, 2022). *Penicillium expansum* can cause significant economic losses in fruit production, mainly apples, pears and peaches. This pathogen produces a secondary metabolite called patulin that can cause acute and chronic toxicity in the human body (Chen et al., 2021). *Botrytis cinerea* is a fungus that infects a wide range of fruits and vegetables and causes a great economic impact. The control of this pathogen is usually done with synthetic fungicides; however, strains resistant to fungicides are increasing and, with the higher demand for residue‐free food, the control of this pathogen is becoming a major challenge (Simone et al., 2021). Among important phytopathogenic bacteria, *Erwinia amylovora* generates huge economic losses mainly in pear orchards and *Xylella fastidiosa* infects a wide list of important crops, such as vineyards, olive groves, almond groves, and citrus orchards. *X. fastidiosa* blocks the hosts' xylemic vessels, making it difficult to absorb water and nutrients, which results in wilting, burning of the marginal and apical area of the leaves, death of branches and, then the entire plant (Landa et al., 2022). In recent years, the excessive use of chemical pesticides to control such pathogens has triggered pathogen resistance, environmental pollution, water pollution and destruction of biodiversity. The use of chemical products has been considered, in recent years, a threat to biodiversity and human and animal health (Omran & Baek, 2022). The impacts of chemical pesticides on human health have been the subject of studies by health professionals and many researchers around the world. The presence of these substances in samples of human blood, breast milk and food has been observed and may be responsible for the onset of diseases such as cancer, mental illness, and various problems related to the reproductive system (Siqueira & Kruse, 2008). This has resulted in increasing pressure to reduce the use of chemical products and make food production more sustainable (Hoarau et al., 2022). For this reason, many chemical products are being withdrawn from the market or some limitations are being imposed on their use. The increasing limitations of plant protection products, combined with the effects of climate change, are causing the emergence of new crop diseases (Daranas et al., 2019). For instance, to control the emergence of phytopathogenic bacteria, copper‐based products are currently the only alternative to control. Nevertheless, these have shown the ability to induce resistance to pathogens, and are toxic to organisms and plants and, as such, in the European Union (EU) its ban is foreseen for the coming years (Daranas et al., 2019). The use of antibiotics in the control of plant pathogens is not allowed in the EU as they may have negative impacts on plant microbiomes and cause the appearance of resistance (Verhaegen et al., 2023).

It is, therefore, essential to search for sustainable alternatives to control pathogens that do not compromise the environment and human health. For this purpose, biological control agents (BCAs) appear among the most promising solutions to the present challenge. It is in our hands to study and explore these biological agents, so that they can be used in plant protection without compromising our future. BCAs are made from living microorganisms, or natural products, that can control pests and diseases, prevent the development of resistance by pathogens, and are considered safe for humans (Hoarau et al., 2022).

When applied to crops, several different outcomes are possible: they can increase plant resistance against infection, and compete for nutrients and space against pathogens. (Köhl et al., 2019), or cause inhibition through the secretion of volatile compounds or by antibiosis (Lengai & Muthomi, 2018). As BCAs may be influenced by biotic and abiotic factors, biological control studies also focus on the study of antimicrobial substances they produce (Daranas et al., 2019). BCAs can be chosen specifically to react to the problematic pathogen and have a very low environmental impact (Mitra et al., 2023). Many studies are being performed to find more suitable BCAs and the European and Mediterranean Plant Protection Organization (EPPO) annually updates a list of BCAs that show no, or acceptable, adverse effects and aims to facilitate decisions on the release of BCAs within EPPO countries (EPPO, 2023). At present, there are 22 bacterial‐based biocontrol agents approved in the EU as biopesticides, 40 fungal‐based and 9 viral‐based (European Commission, n.d.).

Efforts are required to increase available microbial biopesticides for effective plant disease management and that is achieved by searching and studying potential biocontrol agents. In this way, a promising potential biocontrol agents group arises, the lactic acid bacteria (LAB). LAB have shown to produce a variety of compounds that can suppress a wide range of phytopathogens, such as organic acids, bacteriocins, volatile organic compounds (VOCs) and biosurfactants (Gajbhiye & Kapadnis, 2016; Garzón et al., 2017; Narendranath et al., 2001; Sharma & Saharan, 2016). Moreover, antagonistic properties of LAB may also include competition for nutrients (Schnürer & Magnusson, 2005). Another feature that places LAB as promising agents in plant production is their ability to promote plant growth (Abhyankar et al., 2022; Strafella et al., 2021). LAB have been used in food processing and the bioactive substances they produce are widely known. For this reason, they are generally recognized as safe (GRAS) by the Food and Drug Administration (FDA) which exempts them from consuming regulatory approval processes making their commercial application easier (Chen et al., 2021). This review aimed to underline the potential of LAB in sustainable agriculture, focusing on many studies showing their potential as biocontrol agents and as alternatives to chemical usage.

## LACTIC ACID BACTERIA

LAB are Gram‐positive, catalase and oxidase negative and facultative anaerobic bacteria that produce lactic acid as a product of fermentation. LAB belong to the *Lactobacillales* order and contains nine different families (Holzapfel & Wood, 2014). In 2020, a taxonomic reorganization of the *Lactobacillaceae* family created 23 new genera to include organisms previously classified as Lactobacillus, presenting a total of 31 genera, including *Lactobacillus*, *Paralactobacillus*, *Weisella*, *Pediococcus*, *Convivina*, *Leuconostoc* and *Fructobacillus* (Zheng et al., 2020).

LAB are present in environments rich in carbohydrates, including diverse ecological niches such as dairy, fermented foods, water and plants (König et al., 2017). The composition of LAB species in each niche reflects their high adaptation capacity to environmental conditions, which is also dependent on their interactions with other microorganisms, such as antimicrobial activity or competition for nutrients (McAuliffe, 2018). The advances in new sequencing techniques have allowed us to associate specific genetic variations with the adaptation of LAB to specific plant environments (Strafella et al., 2021).

LAB can ferment carbohydrates and produce organic acids as well as antimicrobial compounds such as acetic acid and propionic acid, and they have been used since the dawn of time in the fermentation and preservation of foods (Rodríguez‐Sánchez et al., 2022). Some LAB species only produce lactic acid as the end product and are called homofermentative, and others, in addition to lactic acid, produce acetic acid, ethanol and carbon dioxide and are called heterofermentative (Kanauchi, 2019). The acids produced in the greatest quantity by LAB are lactic acid and acetic acid—both of which are known to have antifungal properties.

The amount and type of organic acids produced by LAB are dependent on factors, such as the species under study, the strain, the culture medium and the growth conditions (Rodríguez‐Sánchez et al., 2022). These acids cause the pH of the medium to decrease which can be hostile to the microorganisms. Organic acids can penetrate the cytoplasmic membrane of microorganisms, leading to intracellular acidification. This action will be dependent on the pH of the medium (Arena et al., 2016). According to Rodríguez‐Sánchez et al. (2022), the concentration of these same acids increases with incubation time in each strain studied. A study has also shown that the inhibitory effect is lost after neutralization of the pH and that the inhibitory effect remains without impact through enzymatic and thermal treatment (Arena et al., 2016).

Today, LAB form part of the most important group of microorganisms for industry. Most LAB possess characteristics that allow them to be used in a wide range of industrial applications due to their tolerance to various stress environments, their simple metabolism and the ability to metabolize numerous carbon sources (Hatti‐Kaul et al., 2018). In the food industry, chemicals have often been used to combat bacteria such as *Staphylococcus aureus*, which—when present in food—grows and produces different enterotoxins that cause food poisoning in humans and animals. These enterotoxins show resistance to acidity and high temperatures. For this reason, it becomes difficult to combat their appearance in food through conventional methods, such as pasteurization. However, this bacterium showed less growth in the medium with low pH levels, thanks to the acidification of the medium resulting from the organic acids secreted by the lactic bacteria (Rodríguez‐Sánchez et al., 2022). LAB have a great capacity to inhibit pathogenic bacteria that contaminate food and cause diseases in humans (Arena et al., 2016). LAB produce bacteriocins which are tolerant to high temperatures and a wide pH range and are colourless and odourless, which makes them suitable for use in the food industry as in food preservation. In addition, they do not cause resistance to pathogens compared to antibiotics since they are of natural origin (Daba & Elkhateeb, 2020). They are also known as probiotics, namely *Lactobacillus* strains of which the effects on human health have been explored where several advantages have been observed, including help in the digestion of specific dietary substrates and increased protection against intestinal infections (Dempsey & Corr, 2022).

In the plant environment, LAB can be found both in the phyllosphere and rhizosphere, as well as in the seeds of many plants (Dayana et al., 2019; Minervini et al., 2015). Glucose, fructose and sucrose are highly found in the phyllosphere and are preferred carbon sources for the fermentative development of LAB (Gao et al., 2019). Among the most frequently found genera on plant tissues are *Lactobacillus*, *Leuconostoc* and *Weisella* (Hatti‐Kaul et al., 2018). In addition, *Lactococcus* and *Streptococcus* from the family *Streptococcaceae* and *Enterococcus* from the family *Enterococcaceae* are also frequently found on plant tissues (Yu et al., 2020).

Many of the characteristics that make LAB one of the most important groups of microorganisms in the transformation and preservation of foods, such as their tolerance to pH and salinity, wide range of growth temperatures, their ability to form biofilms and the production of bioactive compounds (including antimicrobials, antifungal and bacteriocins), are the same that make them great candidates to be used in plant protection and substitute chemicals for sustainable agriculture (Daranas et al., 2019; Simone et al., 2021). In addition, the bioactive compounds LAB produce are very well studied in the food processing industry and have resulted in their designation as GRAS by the US FDA, which accelerates the regulatory approval processes and eases their application in agriculture (Lutz et al., 2012).

Despite LAB–plant interactions not being as well studied as LAB in the food transformation industry, several LAB present in the rhizosphere as well as in other sources have been shown to present antimicrobial properties (Fakri et al., 2018; Fhoula et al., 2013). The carbohydrates released by plant roots contribute to the proliferation of LAB, which breaks down these compounds causing a decrease in the rhizosphere pH and providing a toxic effect on other undesirable microorganisms (Jones, 1998). In addition, metabolites produced by LAB have also been shown to interfere with plant development with the production of plant hormone‐like compounds (Goffin et al., 2010; Sharifi & Ryu, 2018). All these characteristics point to LAB as a new class of Plant Growth Promoting Microbes.

Not all strains of LAB confer protection to plants against pathogens and therefore their selection requires many studies in vitro, ex vivo and in vivo. Furthermore, the classification of the strains used is also an important step in making the requirement for registration of BCA (Daranas et al., 2019). In the next section, we will focus on the roles of LAB as biocontrol agents that show their potential towards sustainable agriculture.

## LAB AS BCAs

LAB have many properties that place them as interesting biocontrol agents, namely due to their ability to produce antimicrobial compounds, to compete with pathogens for nutrients and their role in plant immune response (Gajbhiye & Kapadnis, 2016; Konappa et al., 2016; Peláez et al., 2012; Roselló et al., 2013; Sangmanee & Hongpattarakere, 2014). Antimicrobial compounds produced by LAB can act individually or synergically and include diketopiperazines, 3‐phenylacetate, bacteriocins, hydroxy derivatives of fatty acids, hydrogen peroxide, reuterin, diacetyl and pyrrolidone‐5‐carboxylic acid (Lamont et al., 2017; Siedler et al., 2019). LAB can reduce the growth and spore germination of pathogens, thus decreasing their ability to colonize plants and cause disease. LAB may also act by neutralizing the toxic effects of pathogens or inhibit the production of mycotoxins and reduce post‐harvest decay (Trias et al., 2008; Tsitsigiannis et al., 2012). LAB can also form biofilms, mostly composed of polysaccharides, which provide them with a high antagonistic capacity as well as a high resistance to abiotic stresses (Rezaei et al., 2021).

LAB produce biosurfactants which have antifungal, antiviral and antibacterial activity and may have a role against biofilm formation, motility and pathogenicity (Patel et al., 2021). These molecules, mostly composed of proteins, carbohydrates, lipids and fatty acids, can also facilitate nutrient acquisition and have roles in LAB interaction with the host (Satpute et al., 2016). LAB that have the potential to be used in sustainable agriculture may have an origin in any of the diverse ecological niches they are found, such as dairy, fermented foods, water and plants (König et al., 2017). Several studies are reporting the effective activity of LAB from different sources, as biocontrol agents against many important phytopathogens (Table 1). Studies show that the efficacy of LAB against pathogens differs according to in vivo and in vitro assays, as well as from the different sources that LAB are isolated (Dalié et al., 2010).

**TABLE 1:** Lactic acid bacteria with biocontrol activity on agricultural crops.

LAB	LAB source	Pathogen	In vitro/ex vivo/in vivo	Mechanism/effect suggested	Reference
*Lactococcus lactis* subsp. *lactis*	Fermented milk products (whey)	*Rhizopus stolonifer*	In vitro + reduction or prevention of disease in vivo and in post‐harvest in jackfruit.	Mycelial breakage	Ghosh et al. (2015)
*Lactiplantibacillus plantarum*	Cucumber	*Aspergillus flavus*, *Fusarium graminearum*, *Rhizopus stolonifer*, *B. cinerea*, *Sclerotium oryzae*, *Rhizoctonia solani* and *Sclerotinia minor*	In vitro + cucumber fruits (only *A. flavus*, *F. graminearum*, *R. stolonifer* and *B. cinerea*)	Antifungal compounds	Sathe et al. (2007)
*Latilactobacillus sakei*, *Leuconostoc fallax*, *Lb. plantarum*, *Pediococcus parvulus*, *P. dextrinicus*, *Lb. buchnerii* and *Lactococcus lactis*	Fresh fruits and vegetables and dairy	*Xanthomonas campestris*, *Erwinia carotovora*, *Penicillium expansum*, *Monilinia laxa* and *Botrytis cinerea*	In vitro + apple fruits (only *P. expansum*)	Acidification of medium, organic acids, hydrogen peroxide	Trias et al. (2008)
*Lb. plantarum* and *L. helveticus* (genome shuffling)	Dairy products	*Penicillium digitatum*, *B. cinerea*, *G. cingulate*, *P. citrinum*, *P. roqueforti*, *F. oxysporum*, *A. ochraceus* and *A. niger*	In vitro + kumquat (only *P. digitatum*)	Lactic acid, phenyllactic acid	Wang et al. (2013)
*Pediococcus pentosaceous*	Dairy products (cheese)	*P. expansum*	In vitro + pear, plum and grape fruits	Lactate and phenyllactate	Crowley et al. (2013)
*Lb. plantarum* and *Lacticaseibacillus paracasei*	Plant materials, fermented wheat bran, pickles and sauerkraut	*Penicillium expansum*	In vitro + apple fruit	Antifungal compounds	Matei et al. (2016)
*Levilactobacillus brevis* and *Limosilactobacillus reuteri*	Cheese or porcine gut	*Zymoseptoria tritici*	In vitro and in vivo using wheat seedlings	Antifungal compounds	Lynch et al. (2016)
*Liquorilactobacillus sucicola*, *Weisella paramesenteroides* and *Pediococcus acidilactici*	Orange (fruit, leaves and soil) and peanuts	*P. digitatum*	In vitro + orange fruits	Organic acids, hydrogen peroxide, proteinaceous compounds	Ma et al. (2019)
*Lb. plantarum*	Persian Type Culture Collection (PTCC)	Yeast and moulds	Coating of strawberries for post‐harvest protection	Decrease on pH	Khodaei and Hamidi‐Esfahani (2019)
*Lb. plantarum*, *Lb. pentosus* and *P. pentosaceus*	Steamed cake	*A. niger*, *Cladosporium sphaerospermum* and *P. chrysogenum*	In vitro + pitaya fruit substrate for preservation	Phenolic compounds	Omedi et al. (2019)
*L. plantarum*	Sourdough and tomato	*P. expansum*	In vitro + tomato fruit	Organic acids, phenollic acids, VOCs	Luz et al. (2020)
*Lb. plantarum*	Fermented soybeans	*Aspergillus flavus*	In vitro + fresh maize seeds	Antifungal peptides	Muhialdin et al. (2020)
*Lactiplantibacillus plantarum*	Artisanal sourdoughs	*Botrytis cinerea*	In vitro + kiwi fruits	Lactic acid	De Simone et al. (2021)
*Lactobacillus* sp. and *Lactobacillus acidophilus*	Dairy products (yoghurt and milk)	*Fusarium oxysporum*	In vitro + tomato seeds and tomato seedlings	Antifungal metabolites	Hamed et al. (2011)
*Lacticaseibacillus paracasei*	Soil	*Ralstonia solanacearum*	In vitro + tomato seeds	Induction of plant systemic defence	Konappa et al. (2016)
*Weisella cibaria* and *Lactococcus lactis* subsp. lactis	Papaya seed	*Erwinia mallotivora*	In vitro + papaya plants in nurseries	Organic acids, hydrogen peroxide	Taha et al. (2019)
*Lb. pentosus* and *Leuconostoc fallax*	Fermented Chinese cabbage (*Brassica rapa pekinensis*) and Fermented spicy mustard (*Brassica juncea* (L.) Czern)	*Alternaria brassicicola*, *Xanthomonas campestris*, *pv. campestris* and *Pectobacterium caratovorum*	In vitro + radish slices, cabbage seedlings and detached leaves	Unknown	Lin et al. (2020)
*Lb. plantarum*	Yellow pithaya	*Fusarium fujikuroi*	In vitro	Unknown	Valencia‐Hernandez et al. (2021)
*Lb. plantarum*, *Limosilactobacillus fermentum* and *Lacticaseibacillus paracasei*	Mango	*Colletotrichum gloeosporioides*, *Botryodiplodia theobromae*, *Aspergillus variecolor*, *Aspergillus niger* and *A. flavus*	In vitro + mango fruits	Bioactive compounds	Ranjith et al. (2021)
LAB	Collection of Pure Cultures of Industrial Microorganisms ŁOCK at the Lodz University of Technology, pickled vegetables, milk	*Pectobacterium carotovorum*, *Streptomyces scabiei*, *Alternaria solani*, *Alternaria tenuissima*, *Alternaria alternata*, *Phoma exigua*, *Rhizoctonia solani* and *Colletotrichum coccodes*	In vitro + potato seeds	Organic acids	Steglińska et al. (2022)
*Lb. plantarum* and *Leuconostoc mesenteroides*	Orchard trees (leaves, flowers and fruits), fresh fruits and vegetables from retail markets and ready‐to‐eat commercial products	*Erwinia amylovora*	Detached flowers, leaves and immature pear fruits and apple flowers	Colonization, plantaricin	Roselló et al. (2013)
*Lb. plantarum* and *Leuconostoc mesenteroides*	Cucumber, pear, tomato, cherry and lettuce	*Pseudomonas syringae pv. actinidiae*, *Xanthomonas arboricola pv. pruni* and *Xanthomonas fragariae*	In vitro + kiwifruit plants and plantlets, Prunus plantlets and strawberry plants	pH lowering effect and the production of lactic acid	Daranas et al. (2019)
LAB	Soil and root samples of maize, rye, carrots, garden soils and compost	*Pythium ultimum*	In vitro + cucumber seeds	Unknown	Lutz et al. (2012)
*Lb. plantarum* and *B. amyloliquefaciens*	Silages and forest soil	*Fusarium culmorum and F. graminearum*	In vitro + durum wheat	Organic acids, plantaricin	Baffoni et al. (2015)
*Lactobacillus delbrueckii* subsp. *bulgaricus*, *Leuconostoc mesenteroides* subsp. *dextranicum* and *Lactococcus lactis* subsp. *diacetylactis*	Camel milk	*F. oxysporum*, *F. redolens* and *F. solani*	In vitro + tomato seedlings	Antimicrobial compounds	Zebboudj et al. (2020)
*Lb. plantarum*, *Lacticaseibacillus paracasei* and *Lb. pentosus*	Fermented beverages (Tepache and Tejuino)	*Colletotrichum gloeosporioides*	In vitro	Metabolites	Barrios‐Roblero et al. (2019)
*Lb. plantarum*	Food matrices	*Aspergillus niger*, *Aspergillus flavus*, *Fusarium culmorum*, *Penicillium roqueforti*, *Penicillium expansum*, *Penicillium chrysogenum* and *Cladosporium* spp.	In vitro + fermented oat‐based products	Lactic acid and low pH, phenyllactic acid	Russo et al. (2017)
*Lb. plantarum*	Grape berries	*Aspergillus carbonarius*	In vitro + grape fruits	Acid compounds	Lappa et al. (2018)
*Latilactobacillus sakei*, *Pediococcus acidilactici* and *Pediococcus pentosaceus*	Rye sourdoughs	*Fusarium* spp., *Bipolaris sorokiniana* and *Alternaria* spp.	In vitro + wheat seeds + field assays	Bacteriocin‐like inhibitory substances	Suproniene et al. (2015)

Milk and other dairy products have been used, both fermented with *Lactobacillus* or as a natural source of LAB, as a natural control for powdery mildew on cucurbits (Bettiol & Astiarraga, 1998; DeBacco, 2011; Ferrandino & Smith, 2007). *Lactobacillus* from compost teas have also been shown to be effective against powdery mildew on cucurbits (Naidu et al., 2012).

Many LAB species have shown antifungal activity against *Fusarium graminearum*, the causal agent of Fusarium head blight, a serious fungal disease of cereals; these include *Lactiplantibacillus plantarum*, *Bacillus* species and *Lentilactobacillus buchneri* isolated from corn silage (Paradhipta et al., 2021). In a study performed by Steglińska et al. (2022), *Lactiplantibacillus plantarum* was able to reduce the disease caused by many phytopathogens including *Pectobacterium carotovorum* and *Rhizoctonia solani* but was not able to inhibit *Fusarium oxysporum* and *F. sambucinum* in vivo. In an in vitro assay, López‐Seijas et al. (2019) showed that *Lb. paracasei* and *Lb. plantarum* isolated from wine fermentations were able to reduce the growth of the tomato pathogenic fungus *Fusarium oxysporum* sp. *lycopersici*. Other studies using *Fusarium* species showed that *Lactiplantibacillus plantarum* isolated from pithaya inhibits *Fusarium fujikuroi* growth (Valencia‐Hernandez et al., 2021) and *Lactobacillus delbrueckii* subsp. *lactis* inhibited *Fusarium* species of tomato crown and root rot (Zebboudj et al., 2020). *Lb. plantarum*, as well as *Lb. paracasei* and *Lb. pentosus* has also been shown to decrease the mycelial growth and spore germination of the devastating anthracnose disease, caused by *Colletotrichum gloesporioides*, in papaya (Barrios‐Roblero et al., 2019).

Several studies have also shown LAB as a potential biocontrol agent against the important phytopathogen *Pythium* causing dieback and death in many crops (Lutz et al., 2012). *Lactiplantibacillus plantarum* has also shown antagonism against citrus green rot and *B. cinerea* (De Simone et al., 2021). According to De Simone et al. (2021), 300 strains of LAB were used and tests to inhibit the growth of *B. cinerea* and to characterize the inhibition mechanism were carried out with filtrates from the cultures. In this assay, the LAB showed a weak or moderate ability to inhibit the pathogen under study, whereas 98% of the strains showed no ability to inhibit the growth of *B. cinerea*. Of the 300 strains, only 6 showed a halo of inhibition greater than 10 mm, which means that they have a strong antagonistic ability. To determine the inhibition mechanisms of the six strains of bacteria under study that showed antifungal ability, the filtrates were collected and their pH was measured, where it was found that in all of them, it was below 4 after 24 h and even lower after 48 h. After performing growth inhibition tests with the culture filtrates, it was possible to observe that after 24 h, the filtrates inhibited between 40% and 80% of the pathogen growth. After 48 h, an inhibition of 5%–30% was still observed. These results showed some relationship between the lower pH and the antagonistic activity, leading to the conclusion that those responsible for the inhibition may be the organic acids (De Simone et al., 2021).

In another study reported by Daranas et al. (2019), 55 strains of plant‐associated lactic bacteria were used (*Lb. plantarum*, *Lb. pentosus*, *Leuconostoc mesenteroides*, *Lactococcus lactis* and one unidentified strain). Three pathogenic bacteria were used to test the inhibitory ability of the LAB strains, namely, *Pseudomonas syringae pv.actinidiae*, *Xanthomonas arborica pv.pruni* and *Xanthomonas fragariae*.

Out of 55 strains of LAB under study, 17 strains showed very low activity against *P. syringae pv.actinidiae*, and moderate activity against *X. arborícola pv. Pruni* and moderate to high activity against *X. fragariae*, 33 strains showed moderate or no activity against *P. syringae pv.actinidiae*, moderate to high activity against *X. arborícola pv.pruni* and *X. fragariae* and 5 strains showed generally high activity against all the pathogens under study. Survival of two *Lb. plantarum* strains (PM411 and TC92) on kiwi and strawberry leaves were tested. After inoculation, the population level decreased significantly until Day 5 but remained stable in the following days. The efficacy of strains PM411 and TC92 was compared with other products, namely *Bacillus (B.) subtilis* QST713 and others, where it was found that strain PM411 was effective against *X. fragariae*, which showed a lower development of infections by this pathogen. The TC92 strain did not show significant differences in the fight against *X. arborícola pv.pruni* in comparison with *B. subtilis* QST713. In the fight against *P. syringae pv.actinidiae*, the strain PM411 was able to reduce the incidence of disease in kiwi plants by more than 50%. All this data were obtained from the comparison with the negative control. To characterize the inhibition mechanism, filtrates of the cultures were used in inhibition assays with the pH of the medium without adjustment (pH 3.8) that demonstrated the ability to inhibit the three pathogens under study. This antimicrobial activity was not affected by the culture filtrates when exposed to enzyme treatments; however, it was affected after neutralizing the pH of the culture filtrates. Since enzyme treatments suppressed the antimicrobial activity, the likely culprits responsible for the observed inhibition may be the organic acids secreted by the lactic bacteria (Daranas et al., 2019).

In another study reported by Roselló et al. (2013), 100 strains of LAB that were isolated from leaves, flowers and fruits were used. Bacteria such as *Lb. plantarum* LMG9211, *Lactobacillus delbrueckii subsp. lactis* LMG7930 and *Leuconostoc mesenteroides* CM160 were used. As pathogens *E. amylovora* PMV6076, *P. syringae* EPS94, *E. coli* ATCC5954, *S. aureus* ATCC9144 and *Bacillus subtilis* EPS2000 were used. *B. subtilis* QST713, *Pantoea vagans* C9‐1 and *Pseudomonas fluorescens* EPS62 were used as reference BCAs. In the assays performed, several of the strains under study were found to be able to inhibit the growth of *E. amylovora* compared to the negative control. Of the 100 bacterial strains isolated from leaves, flowers and fruits, 2 (TC54 and TC92) showed very consistent effects in reducing infections. It was also found that some of the bacterial strains (CM209, PM366, PM411, TC54 and TC92) showed not only inhibitory activity against *E. amylovora* but also against *P. syringae*, *E. coli*, *S. aureus* and *B. subtilis*. In this trial, it was also found that strain TC54 significantly reduced the occurrence of *E. amylovora* infections in 100% of the trials. This author also reports that in trials performed on leaves infected with *E. amylovora*, the leaves were treated with strains PM411, TC54 and TC92, where a lower incidence of the disease was observed. Nevertheless, these three strains of LAB showed similar disease‐fighting efficacy as the agent *B. subtilis* QST713 (Roselló et al., 2013). A semi‐field trial was conducted where the three strains, which proved to be potential BCAs, were applied to the flowers of the trees in the field. After 24 h, the plant material was taken to the laboratory and inoculated with *E. amylovora*, and the infection process took place under controlled environmental conditions. In this trial, it was possible to verify that strains TC54 and TC92 significantly decreased the incidence and severity of *E. amylovora* infection. The efficacy of strain TC92 ranged from 78% to 90%, making it the best treatment observed (Roselló et al., 2013).

In another assay (Russo et al., 2017), 88 *Lb. plantarum* strains isolated from various food matrices were used. Fungal pathogens such as *Aspergillus niger*, *Cladosporium* ssp., *A. flavus*, *P. expansum*, *P. chysogenum* and *Fusarium culmorum* were used. A growth inhibition assay of the pathogens was performed on a plate with the *Lb. plantarum* strains in the exponential growth phase and after 5 days, the halo of inhibition was measured and they were classified as mild (when the zone of inhibition was less than 1 mm) or strong (when the zone of inhibition was 1–3 mm). The pathogens *A. niger*, *Cladosporium* ssp. and *A. flavus* were observed as the fungal strains with the most resistance, since between 60% and 80% of the *Lb. plantarum* strains showed no ability to inhibit their growth. However, about 75% of the *Lb. plantarum* strains were able to inhibit the growth of *P. expansum* and *P. chysogenum* and 45% of the strains were able to strongly inhibit *F. culmorum*. Nine strains of *L. plantarum* demonstrated the greatest antifungal properties, where growth inhibition of 50% and 60% of *P. expansum* and *F. culmorum*, respectively, was observed. After verifying the strains with antifungal properties, a new growth inhibition assay was performed with the filtrates from the cell‐free cultures. To characterize the mechanism of inhibition, the filtrates from each culture were subjected to a temperature of 80°C for 10 min and neutralized with 2 M NaOH. The neutralized filtrates were further subjected to enzymatic and heat treatment. Another assay was performed with the filtrates subjected to the above treatments. It was observed that the culture filtrates lost their antagonistic properties only when subjected to pH neutralization, which indicates that those responsible for inhibiting pathogen growth are the organic acids secreted by *Lb. plantarum* (Russo et al., 2017).

LAB have shown antifungal activity against *Zymoseptoria tritici*, the causal agent of septoria leaf blotch in wheat (Lynch et al., 2016). *Pediococcus pentosaceous* and *Weisella confusa* showed antimicrobial activity against several fruit crop pathogens (Crowley et al., 2012a; Crowley et al., 2012b). *Lb. plantarum* and *Lb. pentosus* showed antifungal activity against several filamentous fungi and yeast pathogens (Lipińska et al., 2018). LAB antifungal activity against phytopathogens has also been reported by other authors in many fruit crops and vegetables including pepper, cucumber, kumquat, pitahaya and chilli (Shrestha et al., 2014; Trias et al., 2008).

Seed treatments with LAB have been effectively used to reduce pathogens in wheat and damping off diseases (Hamed et al., 2011). Many studies have shown the biocontrol action of LAB through their ability to neutralize the toxic effects of several pathogens, namely *Fusarium oxysporum* in capsicum (Hamed et al., 2011), in table grapes (Lappa et al., 2018), in wheat and maize (Juodeikiene et al., 2018; Kharazian et al., 2017; Muhialdin et al., 2020). LAB have also shown positive roles in the post‐harvest decay of many fruits and vegetables, presenting preservation properties and being able to increase the shelf life of many products, including cucumber, banana, grapefruits, strawberries, tomato and mango (Fenta & Kibret, 2021; Konappa et al., 2016; Sathe et al., 2007). In addition, the combination of different species of LAB and/or their use together with other substances have revealed synergistic effects on the decrease of diseases caused by several pathogens. As an example, the application of the combination of *Weisella cibaria* and *Lactococcus lactis* in nurseries showed a reduction in the severity of dieback disease in papaya (Dayana et al., 2019; Taha et al., 2019).

The use of *Lb. pentosus* and *Leuconostoc fallax*, isolated from fermented vegetables, together with chitosan showed a decrease in the soft rot disease in radishes caused by *Pectobacterium carotovorum*, on cabbage black spot caused by *Alternaria brassicicola* and black rot caused by *Xanthomonas campestris* (Lin et al., 2020). In addition, the addition of divalent cations such as Ca^2+^ and Mg^2+^ in the culture medium increased the antifungal activity of three different strains of *L. delbrueckii* against *Aspergillus flavus*, *Trichoderma viride*, *Penicillium* sp. and *Geotrichum candidatum* (Matevosyan et al., 2020).

The combination of LAB with carboxymethyl cellulose coatings improved the shelf life of strawberries by reducing the growth of yeast and fungi (Khodaei & Hamidi‐Esfahani, 2019). In addition, the combination of *Lb. plantarum* with a polysaccharide from *W. confusa* and its use as an edible coating on cherry tomato showed antifungal activity against *Fusarium* sp. and *Rhizopus stolonifera*, and was able to control weight loss and slow respiration rate while maintaining firmness of the fruit (Álvarez‐Satizabal et al., 2021).

The biocontrol activity of LAB has also been reported for nematodes and insects. For example, LAB enclosing poly (ε‐caprolactone) microcapsules have been shown to promote higher lactic acid production and enhance the viability of LAB cells and have been used to remove root‐knot nematodes in horticultural crops (Takei et al., 2008). In addition, metabolites produced by *Latilactobacillus sakei* and *Latilactobacillus curvatus* have been shown to present nematocidal capacity (Kim & Jazwinski, 2018). The LAB *Oenococcus oeni* has been shown to release metabolites that attract the fruit fly *Drosophila suzukii*, suggesting its potential use as a bait enhancer, resulting in a high capture rate in traps (Alawamleh et al., 2021). Further studies are needed to explore these metabolites produced by LAB which may have application in the monitoring of insect pests.

## LIMITATIONS AND CHALLENGES

Despite the many studies on the potential of LAB as biocontrol agents in agriculture, there is still a lack of LAB‐based biocontrol agents registered as biopesticides. Many reasons limit the commercialization of LAB‐based products. One of the aspects is that most antagonistic tests with LAB have been performed in vitro and under controlled environments and field experiments are scarce. In fact, and as happens with other BCAs, when these microorganisms are tested in the field, their capacity to survive and to produce compounds to control pathogens is usually greatly reduced, dependent on environmental conditions like temperature, humidity, as well as nutrient availability, microbial communities present and host nature to name a few (Bonaterra et al., 2022). This reduction of efficacy is even more notable in the case of non‐native species, which have more difficulty in adapting to new habitats (Tabassum et al., 2024). This is one of the main challenges: to ensure that LAB can survive and maintain the bioactivity in the field. This can also be achieved by developing effective bioformulations for the application of LAB in the field that will favour functional implantation of LAB in the field; this can include the selection of specific strains more adapted to the phytomicrobiome, the development of protective carriers and the continuous application of LAB to maintain the sufficient number of viable cells. Another alternative could be to isolate and purify LAB bioactive compounds and apply them directly to crops (Maki et al., 2021), as already done for other BCAs (Gray et al., 2006). Nanotechnology is a promising field in many areas and agriculture is no exception (Cruz‐Luna et al., 2021). The use of LAB in nanotechnology has already shown promising results with the control of *Fusarium culmorum* and *Fusarium graninearum* using biological selenium nanoparticles synthesized by *Lactobacillus acidophilus* (El‐Saadony et al., 2021). It is imperative to gather all the investigations on LAB, focusing on their mechanisms of action and developing strategies to increase their activity in the field.

## CONCLUDING REMARKS

Agriculture faces urgent challenges such as climate change, emerging pathogens, the need to reduce chemical pesticide use, and a growing global population. This review highlights the high potential of LAB as biocontrol agents for important plant pathogens, making them promising candidates to replace synthetic chemicals and achieve food security for sustainable agriculture. LAB have a long history in food science and hold GRAS status, making them suitable for plant protection applications. However, LAB still face limitations and challenges in agricultural use, requiring further studies on their biocontrol efficiency in the field and interactions with various abiotic and biotic conditions. Additionally, exploring different forms of LAB bioproduction to reduce costs and developing effective formulations are essential steps before commercial development.

## AUTHOR CONTRIBUTIONS

**Andreia Saragoça:** Writing – original draft. **Henrique Canha:** Writing – review and editing. **Carla M. R. Varanda:** Supervision; writing – review and editing. **Patrick Materatski:** Supervision; writing – review and editing. **Ana Isabel Cordeiro:** Supervision; review and editing. **José Gama:** Supervision; review and editing.

## CONFLICT OF INTEREST STATEMENT

The authors declare no conflicts of interest.

## Data Availability

Data sharing not applicable to this article as no data sets were generated or analysed during the current study.
